# Staphylococcal Corneocyte Adhesion: Assay Optimization and Roles of Aap and SasG Adhesins in the Establishment of Healthy Skin Colonization

**DOI:** 10.1128/spectrum.02469-22

**Published:** 2022-10-11

**Authors:** Krista B. Mills, Paroma Roy, Jakub M. Kwiecinski, Paul D. Fey, Alexander R. Horswill

**Affiliations:** a Department of Immunology and Microbiology, University of Colorado Anschutz Medical Campus, Aurora, Colorado, USA; b Department of Pathology and Microbiology, University of Nebraska Medical Center, Omaha, Nebraska, USA; c Department of Microbiology, Faculty of Biochemistry, Biophysics and Biotechnology, Jagiellonian University, Krakow, Poland; d Department of Veterans Affairs, VA Eastern Colorado Healthcare System, Aurora, Colorado, USA; University of North Dakota

**Keywords:** *Staphylococcus*, corneocytes, adhesion, fluorescence microscopy, accumulation-associated protein, Aap, surface protein G, SasG

## Abstract

Staphylococcus aureus is an opportunistic pathogen that causes the majority of wound and soft tissue infections. The accumulation-associated protein (Aap) from S. epidermidis and surface protein G (SasG) from S. aureus are cell wall-anchored (CWA) proteins known to be important in adhesion to healthy corneocytes from human skin. We investigated the mechanisms by which S. aureus colonizes healthy human skin by developing an optimized corneocyte adhesion assay. Trypan blue was used for enhanced red autofluorescent visualization of corneocytes with an overlay of green-fluorescent bacteria. The percent area of bacterial adhesion for images acquired by a fluorescence microscope was quantified using Fiji ImageJ. Using this optimized imaging procedure, differences in adhesion between various species and strains of staphylococci were measured. The ability of purified SasG to reduce Staphylococcus epidermidis adhesion was investigated in order to determine if these CWA proteins can compete for binding sites. To further test CWA-mediated adhesion, we engineered a nonadhering *S. carnosus* strain to express full-length SasG from two methicillin-resistant S. aureus (MRSA) strains. Finally, we demonstrated that the SasG A domain was a critical region of this surface protein for adherence to healthy human corneocytes. The developed imaging and expression methods are useful for studying staphylococcal adhesion to healthy human skin and have the potential to be used with a wide variety of fluorescently labeled organisms on both healthy and disease-state (such as atopic dermatitis) corneocytes.

**IMPORTANCE** The skin is the largest organ of the human body and acts as a shield against hazards such as harmful bacteria like Staphylococcus aureus. A diverse skin microbiota and immune cross talk control S. aureus numbers. S. aureus can bind to healthy skin and subsequently proliferate when the skin barrier is compromised, such as in a wound or in patients with atopic dermatitis (AD). It is important to understand these mechanisms in an effort to prevent pathogenic bacteria from causing infection. We describe an augmented corneocyte adhesion assay using fluorescence microscopy to study binding of various staphylococcal species to healthy human skin cells. In addition, we tested the ability of homologous proteins from different staphylococcal species to reduce binding, and developed a new *S. carnosus* expression system to test individual protein binding properties. Our newly developed methods and findings will enhance the understanding of how staphylococci bind to healthy human skin.

## INTRODUCTION

Staphylococcus aureus is a virulent, opportunistic pathogen that is a significant burden on hospital and community health (1). Skin adherence is a critical component of S. aureus pathogenesis, as transient skin colonization is thought to be a key step in the transition to infection (2). This Gram-positive pathogen is the cause of up to 76% of all recorded cases of skin and soft tissue infections (3, 4). S. aureus colonization is asymptomatic and transient, with approximately 30% of humans permanently colonized in the anterior nares (5, 6). In contrast, skin colonization occurs at rates of 5% or less (7–10). Epidermal homeostasis is maintained by environmental factors and commensal microbiota/immune system cross talk that control S. aureus numbers, contributing to the low colonization rate (7, 11–16). It is important to study bacterial adhesion mechanisms and cross-interactions between different species on the skin and the reservoir nasal epithelia, considering that skin and soft tissue infections can lead to further metastatic infection (7, 16, 17).

The skin is the largest organ in the human body and acts as an essential barrier against S. aureus invasion and infection (18). The skin is composed of three layers: the epidermis as the outermost layer, the dermis, and the hypodermis as the innermost layer (19). The most superficial region of the epidermis is the stratum corneum (SC), which is formed by terminal differentiation of keratinocytes upward through the epidermal layers into corneocytes (denucleated keratinocytes with cornified envelopes in replacement of membranes) (18). As the outermost layer, the SC is the first site of contact for S. aureus, and attachment is a crucial prerequisite for infection. S. aureus utilizes an array of cell wall-anchored (CWA) proteins to mediate initial attachment to the skin, which is essential for colonization (5, 20, 21). Different CWA proteins are utilized depending on whether the skin is healthy or compromised. For example, clumping factor B (ClfB) has been demonstrated to be important for S. aureus adhesion to atopic dermatitis (AD) corneocytes (22). For healthy corneocytes, recent studies have indicated that S. aureus surface protein G (SasG) and the homologous protein in Staphylococcus epidermidis, accumulation-associated protein (Aap), are important for adhesion to both human skin and nasal corneocytes (23–26).

Corneocyte adhesion assays have been characterized previously for use in studying adhesion of staphylococci to healthy animal and human corneocytes (25, 27–30) and to corneocytes from the nose and AD skin (22, 26). This article builds and improves upon these previous works by describing an optimized corneocyte adhesion assay using fluorescence microscopy to provide enhanced qualitative and quantitative analysis of staphylococcal adhesion to healthy human corneocytes. Using this new method, the role of SasG in S. aureus adhesion to healthy human corneocytes was further explored. The ability of recombinant protein to block staphylococcal adhesion by blocking the ubiquitous colonizer S. epidermidis with SasG is demonstrated. Additionally, a Staphylococcus carnosus strain was engineered for heterologous protein expression to study the function of SasG-mediated adhesion. In S. epidermidis, Aap-mediated adhesion is dependent on the conserved L-type lectin subdomain within the N-terminal A domain (25), and as SasG is homologous to Aap, the importance of the A domain in SasG-mediated adhesion was explored. Collectively, this optimized assay and new tools were used to demonstrate an important role of SasG and Aap in S. aureus and S. epidermidis binding of human corneocytes and identify a key function for the A domain in these interactions.

## RESULTS

### Optimization of corneocyte staining.

In order to acquire images in which corneocytes fluoresced in a contrasting color against the green-fluorescing bacteria, a variety of methods and dyes were selected. Antibody staining with an anti-keratin 14 rabbit primary monoclonal antibody (Sigma-Aldrich) and Alexa Fluor 594 anti-rabbit IgG (Invitrogen) secondary antibody was attempted first (data not shown). This method did not result in clear images, and the images autofluoresced red, green, and blue when imaged with the respective color channels on the fluorescence microscope. A variety of dyes were then tested for either red or blue autofluorescence without autofluorescing green (data not shown). Three hundred microliters of the following dyes was incubated on corneocytes for 15 min at room temperature and imaged with the red, green, and blue channels on the fluorescence microscope: safranin, methyl green, Congo red, orange G, rhodamine B, fluorescein green, and trypan blue. Of these dyes, trypan blue (which stains dead cells) was the only dye that did not autofluoresce green and resulted in clear images with the red channel.

**FIG 1:**
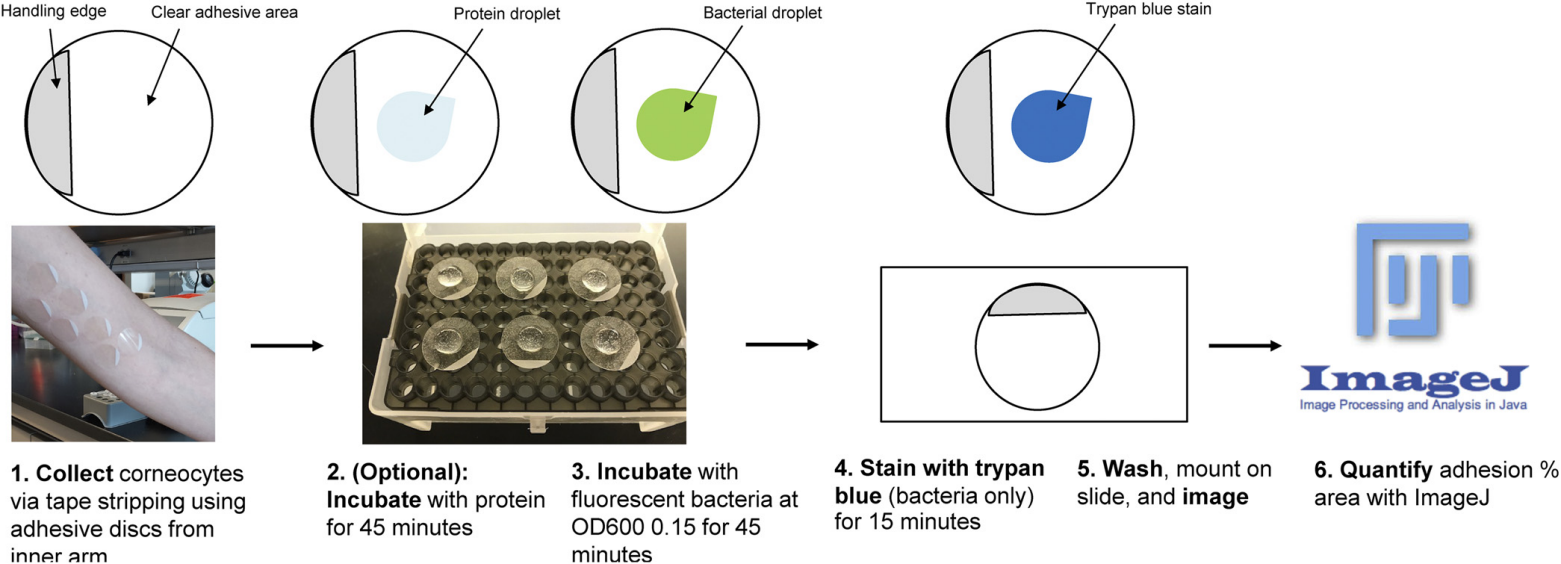
Schematic of the corneocyte adhesion assay. (Step 1) Corneocytes are collected with tape stripping discs from the upper or lower arm near the elbow of a volunteer with healthy skin. (Step 2) Corneocytes can be preincubated with protein for 45 min or (step 3) incubated with fluorescent bacteria at an OD_600_ of 0.15 for 45 min. (Step 4) Corneocytes incubated with bacteria only are stained with trypan blue for 15 min. (Step 5) After incubation and/or staining, corneocytes are washed, mounted on a slide, and imaged with a fluorescence microscope. (Step 6) Bacterial images are quantified for percent area of adhesion with Fiji ImageJ.

**FIG 2:**
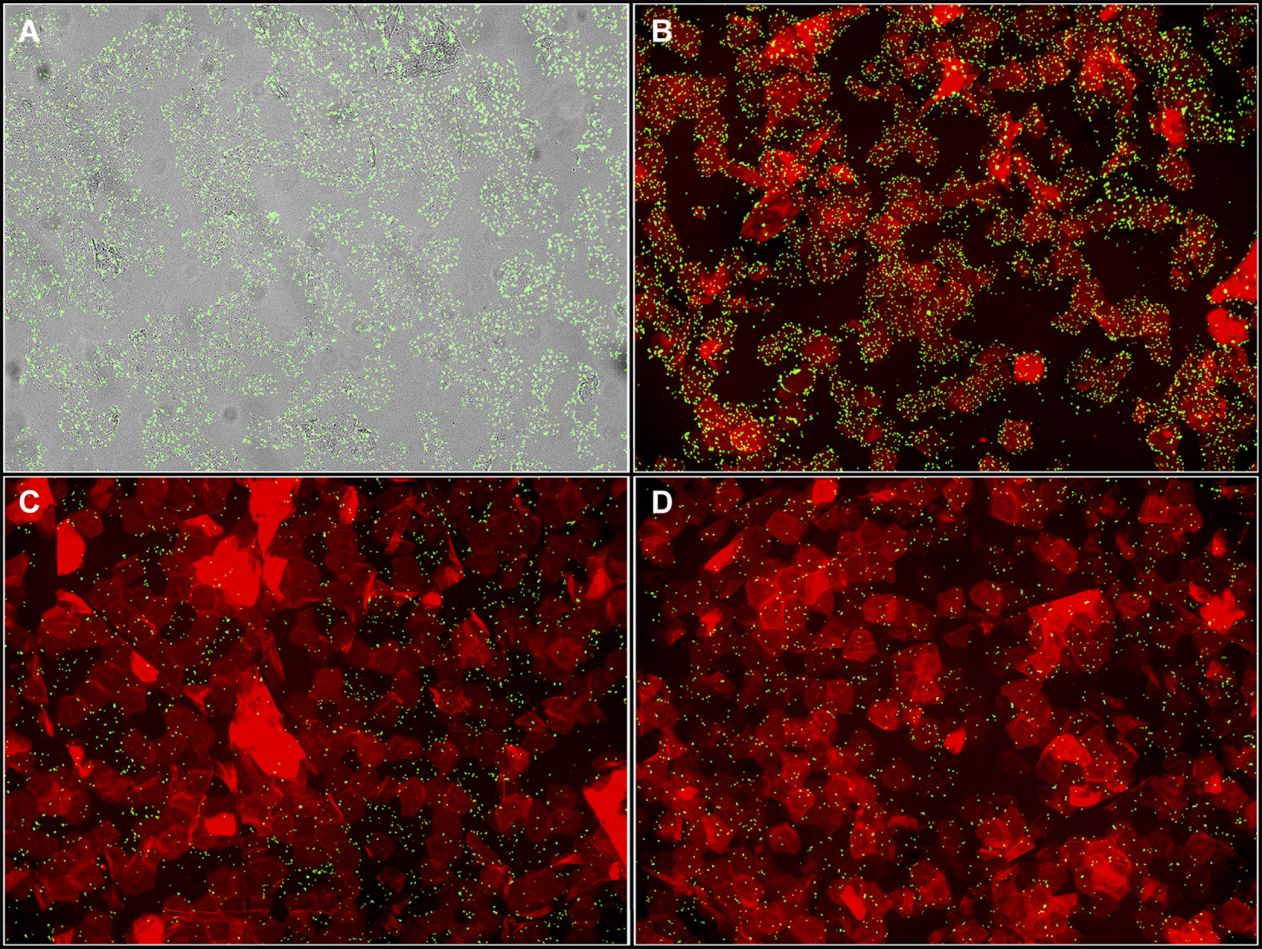
Optimization of corneocyte red autofluorescence with trypan blue. Corneocytes were incubated with (A) S. epidermidis Δ*ica* only, (B) bacteria first followed by trypan blue, (C) trypan blue first followed by bacteria, or (D) a mixture of 300 μL bacteria and 300 μL trypan blue at the same time.

Autofluorescent staining of corneocytes with trypan blue and incubation with green fluorescent protein (GFP)-expressing bacteria was then optimized. Corneocyte collection and incubation with S. epidermidis Δ*ica* were performed as described in Materials and Methods (Fig. 1). Four conditions were tested on corneocytes: a bacteria-only control (Fig. 2A), incubation with bacteria followed by incubation with trypan blue (Fig. 2B), incubation with trypan blue for 15 min followed by incubation with bacteria (Fig. 2C), and mixing 300 μL of prepared bacterial culture and 300 μL of trypan blue and then incubating the entire mixture on corneocytes for 45 min (Fig. 2D). Incubating the corneocytes with bacteria first followed by trypan blue (Fig. 2B) resulted in the image that most closely resembled the bacteria-only control. Incubating corneocytes with trypan blue followed by bacteria resulted in lower bacterial numbers and less nonspecific binding than the bacteria-only control (Fig. 2C). Incubating corneocytes with a 600-μL total mixture of prepared bacterial culture and trypan blue also resulted in lower bacterial numbers than the bacteria-only control and some nonspecific binding (Fig. 2D). As a result, the corneocyte staining technique chosen was that depicted in Fig. 2B, as this technique did not result in a loss of bacterial numbers or nonspecific binding.

### Imaging and quantifying adhesion of staphylococci to corneocytes.

**FIG 3:**
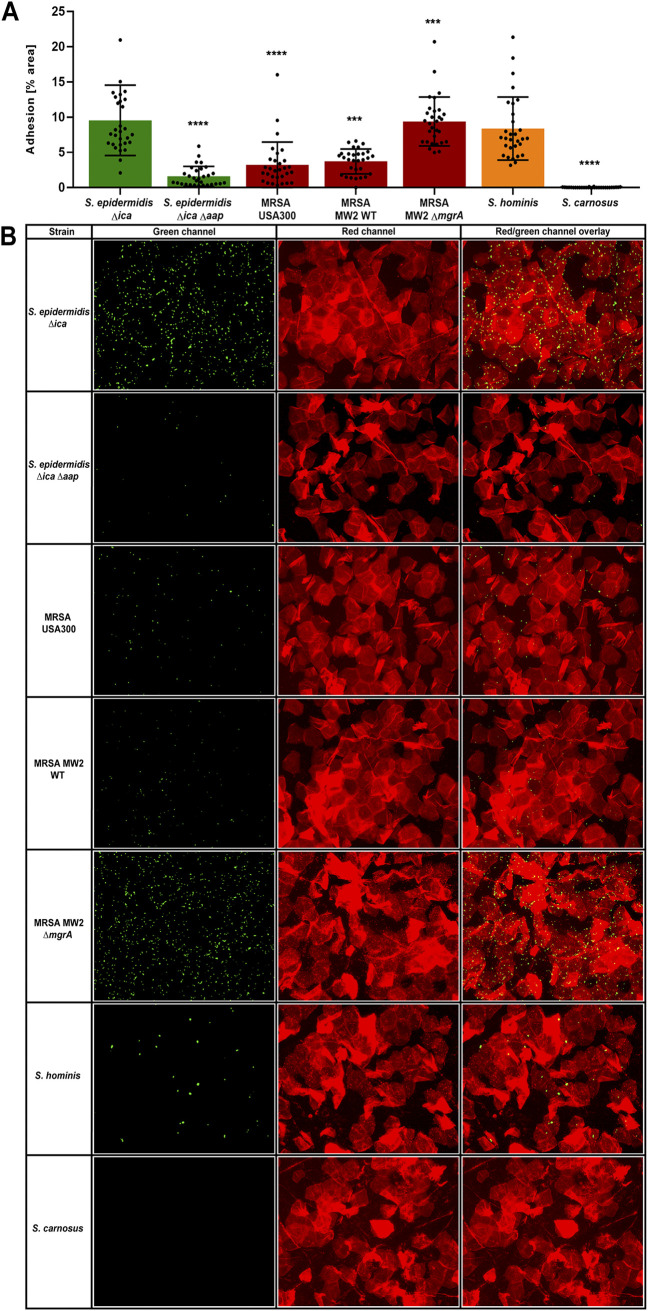
Adhesion of various staphylococci to corneocytes. Various staphylococcal species and strains were tested for adhesion to healthy human corneocytes: S. epidermidis Δ*ica* (AH2983), S. epidermidis Δ*ica* Δ*aap* (AH2984), MRSA USA300 (AH3669), MRSA MW2 WT (AH4477), MRSA MW2 Δ*mgrA* (AH4478), S. hominis (AH5037), and *S. carnosus* (AH5687). (A) The percent area of adhesion in 10 images from three independent experiments (*n* = 30) was measured with Fiji ImageJ and analyzed in GraphPad Prism. Statistical significance was analyzed using the Kruskal-Wallis nonparametric test. Statistical significance of all groups except for MRSA MW2 Δ*mgrA* are mapped to S. epidermidis *Δica* (***, *P = *0.0005; ****, *P < *0.0001). Statistical significance of MRSA MW2 Δ*mgrA* is mapped to MRSA MW2 WT (***, *P = *0.0001). (B) Representative green-channel, red-channel, and red- and green-channel overlay images of each species and strain tested in the corneocyte adhesion assay.

A variety of staphylococcal strains (Table 1) were tested for the ability to adhere to healthy human corneocytes. S. epidermidis *Δica* and S. epidermidis *Δica Δaap* have already been tested for adhesion to corneocytes by Roy et al. (25) and were used as controls. As shown by Roy et al. (25), polysaccharide intercellular adhesin (PIA) does not contribute to corneocyte adhesion, and therefore, the Δ*ica* strain was used as a wild-type (WT) background in this study. In support of these findings, S. epidermidis *Δica Δaap* exhibits significantly less adhesion to corneocytes than S. epidermidis *Δica* (Fig. 3A). S. aureus is infrequently isolated from healthy skin (9), and both USA300 methicillin-resistant S. aureus (MRSA) strain LAC and USA400 MRSA strain MW2 exhibited significantly less adhesion to corneocytes than S. epidermidis (Fig. 3A). Roy et al. (25) also observed that MW2 *ΔmgrA* exhibits significantly greater adhesion to corneocytes than MW2 WT, and we confirmed these findings (Fig. 3A).

**TABLE 1:** Bacterial strains used in this study^*a*^

Strain	Species	Derivative strain	Genotype	Plasmid
AH2983	S. epidermidis	1457	Δ*ica*	pCM29
AH2984	S. epidermidis	1457	Δ*ica* Δ*aap*	pCM29
AH3669	MRSA	LAC (USA300)	NA	pCM29
AH4477	MRSA	MW2 (USA400)	NA	pCM29
AH4478	MRSA	MW2 (USA400)	Δ*mgrA*	pCM29
AH5037	S. hominis	C5	NA	pCM29
AH5687	*S. carnosus*	TM300	Δ*SCA_2238*::sGFP Δ*SCA_2092*	NA
AH5905	*S. carnosus*	TM300	Δ*SCA_2238*::sGFP Δ*SCA_2092*	pALC2073-*sasG*_MW2_
AH5907	*S. carnosus*	TM300	Δ*SCA_2238*::sGFP Δ*SCA_2092*	pALC2073
AH5908	*S. carnosus*	TM300	Δ*SCA_2238*::sGFP Δ*SCA_2092*	pALC2073-*sasG*_COL_
AH6012	*S. carnosus*	TM300	Δ*SCA_2238*::sGFP Δ*SCA_2092*	pHC109 (pALC2073-*sasG*_MW2ΔA_)

a NA, not applicable.

Staphylococcus hominis is one of the coagulase-negative staphylococci (CoNS) most frequently isolated from the skin (31) and, as expected, adheres to corneocytes similarly to S. epidermidis Δ*ica* (Fig. 3A). *S. carnosus* TM300 is an avirulent species that lacks many surface adhesins (32). In support of this, we observed that this species does not adhere to corneocytes (Fig. 3A). Representative green-channel, red-channel, and red- and green-channel overlay images of each strain in this assay can be seen in Fig. 3B.

### Purified SasG blocks adhesion of S. epidermidis to corneocytes.

**FIG 4:**
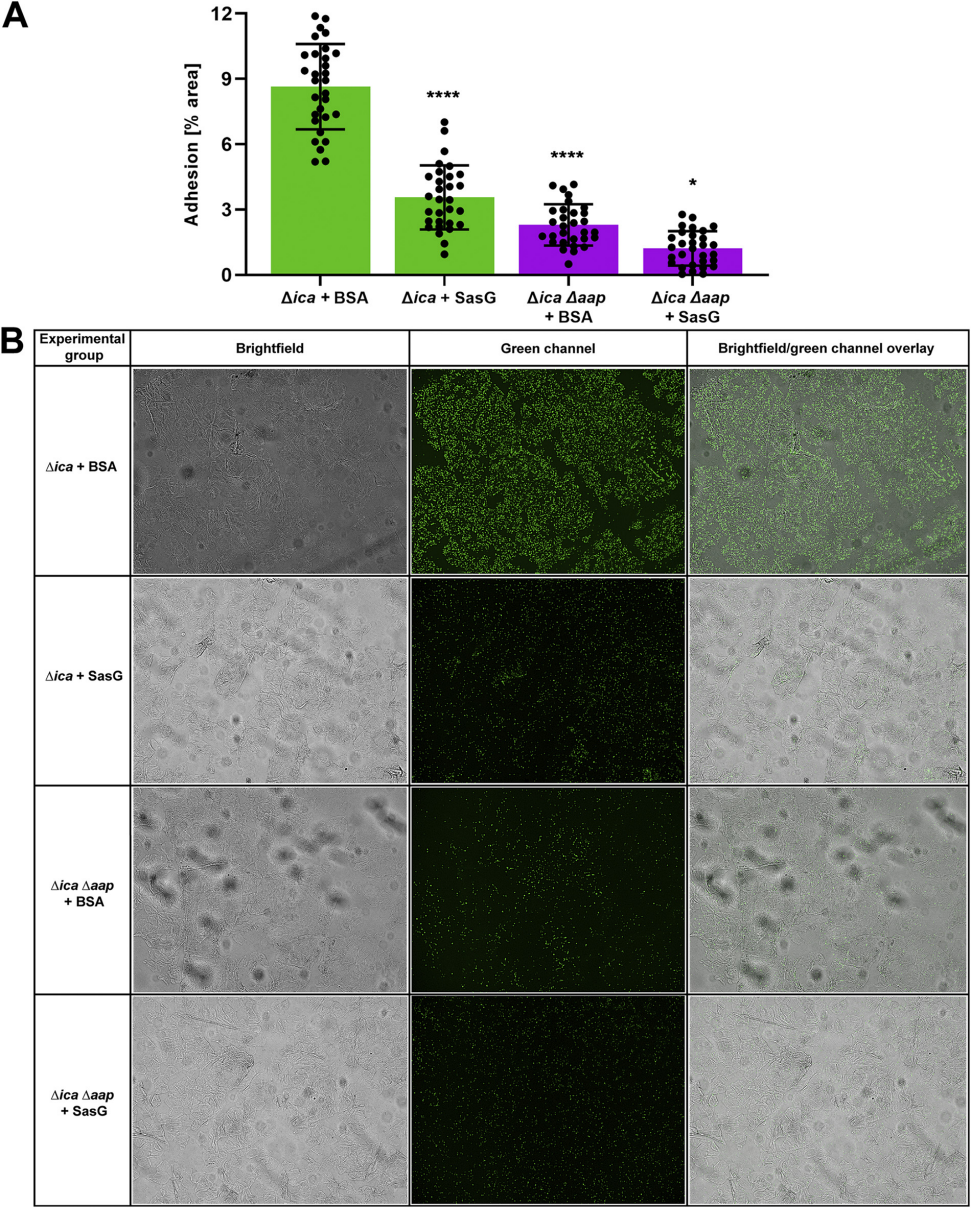
Blocking adhesion of S. epidermidis to corneocytes with SasG. (A) S. epidermidis 1457 Δ*ica* (AH2983) and S. epidermidis 1457 Δ*ica* Δ*aap* (AH2984) were tested for adhesion to healthy human corneocytes after incubation/blocking with full-length SasG. Corneocytes were incubated with 300 μL BSA as a protein control. The percent area of adhesion in 10 images from three independent experiments (*n* = 30) was measured with Fiji ImageJ and analyzed in GraphPad Prism. Statistical significance was analyzed using one-way ANOVA. Statistical significance of all groups except for the Δ*ica* Δ*aap* strain plus SasG are mapped to the Δ*ica* strain plus BSA (*P < *0.0001). Statistical significance of the Δ*ica* Δ*aap* strain plus SasG is mapped to the Δ*ica* Δ*aap* strain plus BSA (*P = *0.0447). (B) Representative bright-field, green-channel, and bright-field/green-channel overlay images of BSA and SasG experimental groups tested in the corneocyte adhesion assay.

The ability of protein to block bacterial adhesion in our assay was then tested. Many strains of S. aureus express a protein homologous to Aap called SasG (24–26). We had previously purified and characterized full-length recombinant SasG (33). While the host ligands of Aap and SasG have yet to be elucidated, these surface proteins share up to 95% sequence identity in the A domain (25), suggesting that they may bind to the same host ligand and compete with other staphylococci that possess homologous proteins. The ability of purified SasG to inhibit adhesion of S. epidermidis 1457 Δ*ica* and the Δ*ica* Δ*aap* double mutant to corneocytes was tested. Incubating corneocytes with 100 μg/mL of SasG resulted in a significant decrease in adhesion of S. epidermidis Δ*ica* compared to the bovine serum albumin (BSA) control, demonstrating the ability of a protein homologous to Aap to block access of S. epidermidis to the host ligand (Fig. 4A). Incubating corneocytes with 100 μg/mL of SasG also resulted in a very slight decrease of adhesion of S. epidermidis Δ*ica* Δ*aap* to corneocytes, but this decrease was not significantly different from the BSA control (Fig. 4A). Representative bright-field, green-channel, and bright-field/green-channel overlay images can be seen in Fig. 4B. These images were not taken with red autofluorescence of corneocytes via trypan blue due to an overall lower abundance of bacteria than in nonstained controls (data not shown).

### SasG expression promotes *S. carnosus* adhesion to corneocytes.

*S. carnosus* is an avirulent and apathogenic CoNS lacking many virulence factors present in other species, such as S. epidermidis and S. aureus (34). We constructed strain AH5687 as a new tool to assess the function of bacterial surface proteins. To reduce any background binding, allelic replacement was used to inactivate genes with homology to S. aureus ClfB (SCA_2283) and protein A (SCA_2092), and we also inserted GFP into the SCA_2283 locus to aid microscopy visualization. As shown in Fig. 3, *S. carnosus* strain AH5687 does not adhere to human corneocytes naturally and therefore is an ideal host for heterologous expression of SasG or other surface proteins.

**FIG 5:**
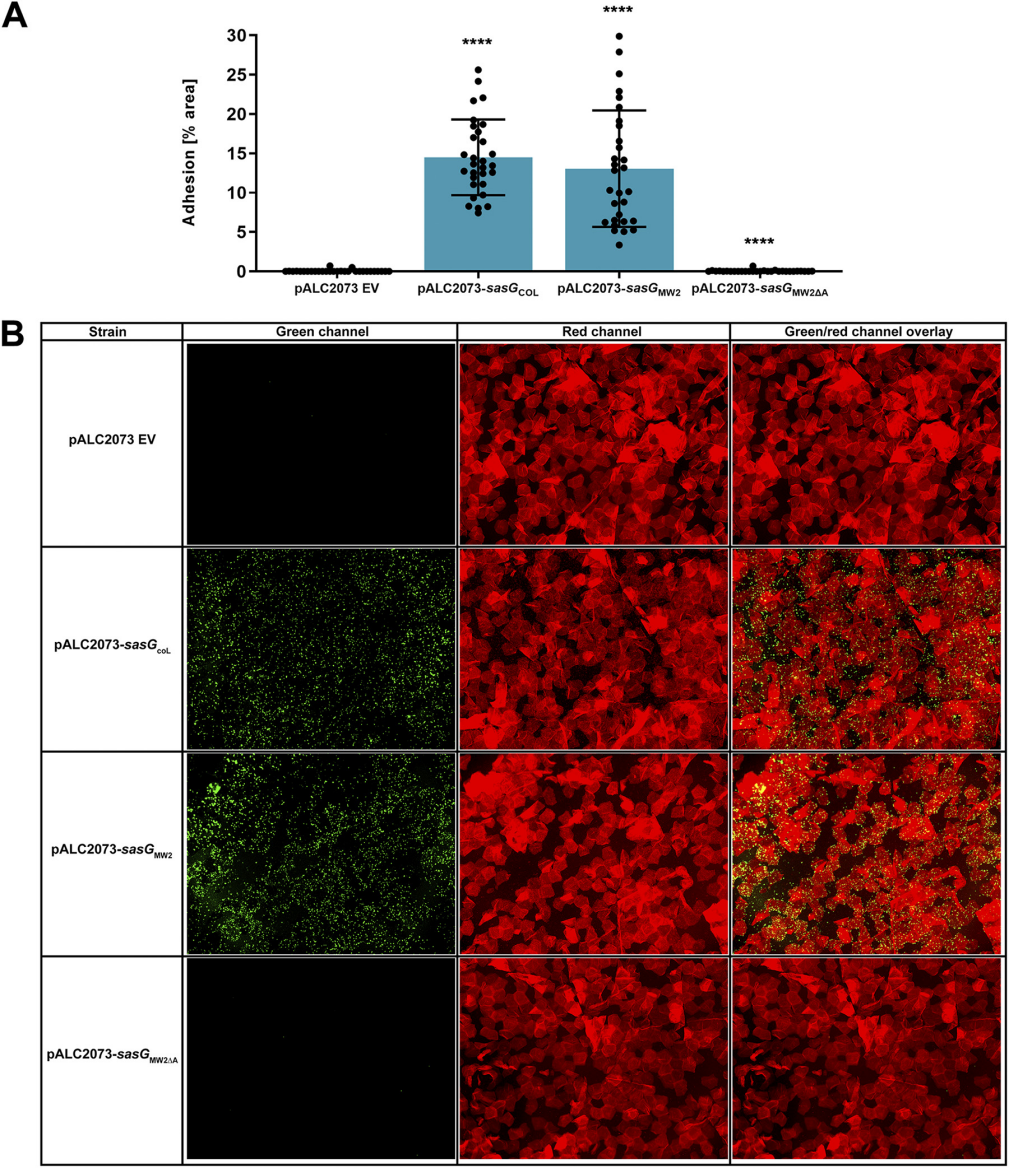
SasG expression promotes *S. carnosus* adhesion to corneocytes. (A) pALC2073 empty vector (EV), full-length SasG-expressing strains AH5908 (pALC2073-*sasG*_COL_) and AH5905 (pALC2073-*sasG*_MW2_), and AH6012 (pALC2073-*sasG*_MW2ΔA_) were tested for the ability to adhere to healthy human corneocytes. The percent area of adhesion in 10 images from three independent experiments (*n* = 30) was measured with Fiji ImageJ and analyzed in GraphPad Prism. Statistical significance was analyzed using the Kruskal-Wallis nonparametric test. Statistical significance of all groups except for pALC2073-*sasG*_MW2ΔA_ is mapped to pALC2073 EV (*P < *0.0001). Statistical significance of pALC2073-*sasG*_MW2ΔA_ is mapped to pALC2073-*sasG*_MW2_ (*P < *0.0001). (B) Representative green-channel, red-channel, and red- and green-channel overlay images of each species and strain tested in the corneocyte adhesion assay.

We hypothesized that expression of only SasG would enable attachment of *S. carnosus* to human corneocytes. As an expression vector, we used pALC2073, a known tetracycline-inducible plasmid with high background expression (35). We selected two strains that express full-length SasG, including MRSA strain COL and USA400 MRSA strain MW2. pALC2073-*sasG*_MW2_ was previously constructed (33), and we cloned pALC2073-*sasG*_COL_ and electroporated these plasmids into *S. carnosus* strain AH5687. *S. carnosus* containing just pALC2073 did not adhere to corneocytes (Fig. 5A), confirming the data shown in Fig. 3. However, *S. carnosus* containing either pALC2073-*sasG*_COL_ or pALC2073-*sasG*_MW2_ adhered strongly to corneocytes, indicating full-length SasG from both of these strains is fully capable of adhesion (Fig. 5A). Representative green-channel, red-channel, and red- and green-channel overlay images of each strain in this assay can be seen in Fig. 5B. For each pALC2073 plasmid construct, tetracycline induction was not necessary to obtain significant adhesion to corneocytes.

To expand on these findings, we tested the contribution of the SasG A domain to adherence. As shown by Roy et al. (25), the Aap A domain is important for adhesion to human skin corneocytes, and we reasoned that SasG-mediated adhesion would also be dependent upon the A domain. We therefore constructed pALC2073-*sasG*_MW2ΔA_, which encodes SasG lacking the A domain, and electroporated this plasmid into *S. carnosus* strain AH5687. *S. carnosus* containing pALC2073-*sasG*_MW2ΔA_ did not adhere to corneocytes and exhibited adherence levels similar to those seen with pALC2073 EV (Fig. 5A). Microscopy imaging of these corneocytes confirmed this observation (Fig. 5B). To verify SasG expression, SDS-PAGE electrophoresis and Coomassie staining of proteins from the cell wall of *S. carnosus* strains AH5905 (pALC2073-*sasG*_MW2_), AH5907 (pAL2073 EV), AH5908 (pALC2073-*sasG*_COL_), and AH6012 (pALC2073-*sasG*_MW2ΔA_) grown in liquid culture and solubilized by lysostaphin was conducted (Fig. S1). Strains were grown either with or without induction with 100 ng/mL anhydrotetracycline. SasG from strain MRSA MW2 was characterized previously to be ~230 kDa (36), with a predicted molecular mass of 150 kDa, while the predicted molecular mass of SasG from strain MRSA COL is similar to that of MRSA MW2 at 136 kDa. Coomassie staining verified the previously characterized molecular mass, with corresponding bands for AH5905 and AH5908 at ~230 kDa (Fig. S1). A band at ~230 kDa was missing from AH6012, with the next most prominent band at ~125 kDa, verifying a lower molecular mass corresponding to deletion of the SasG A domain from MRSA MW2 (Fig. S1). A band at ~230 kDa was also missing from AH5907, with the next most prominent band at ~75 kDa, verifying the lack of SasG expression on the EV plasmid. There were no differences in SasG expression between uninduced strains and those induced with anhydrotetracycline (Fig. S1). These findings indicate that multiple SasG variants are capable of and important for adherence to corneocytes, which is dependent upon the A domain. Finally, we demonstrated that our newly constructed *S. carnosus* strain, AH5687, is a useful tool for heterologous expression of bacterial surface proteins.

## DISCUSSION

Corneocyte adhesion assays have been characterized in a wide variety of applications for studying bacterial adhesion to both human and animal corneocytes (25, 27–30). However, in past studies, microscopy images of corneocytes have largely been limited to using only the brightfield channel, which reduces the clarity. While staphylococcal adhesion methods to human corneocytes have been developed (25), we endeavored to improve on this assay to better visualize the human host cells. Our new method allows superior images of corneocytes to be acquired via red autofluorescence with trypan blue, a dye that is commonly used for cell culture applications to stain dead cells. To the best of our knowledge, this method is the first to combine trypan blue staining with overlaying of green-channel images to study adhesion of staphylococci to corneocytes. Additionally, we engineered a new *S. carnosus* expression strain as a heterologous host to study staphylococcal adhesion to human corneocytes. As the skin is the largest organ of the human body and acts as an essential barrier against a wide array of pathogens, especially those of the genus Staphylococcus (7, 37), the optimized assay and tools described in this work are relevant to the study aspects of both microbial colonization and adhesion to healthy human skin.

This optimized method expanded on the importance of SasG to S. aureus adhesion to healthy human corneocytes. We confirmed the findings by Roy et al. (25) that loss of Aap abrogates S. epidermidis binding to corneocytes and mutation of MgrA increases SasG expression and subsequent MRSA MW2 adhesion to corneocytes. We demonstrated the ability of purified SasG to reduce adhesion to corneocytes of other staphylococci and showed that expressing full-length SasG from two MRSA strains in *S. carnosus* enabled a nonadherent staphylococcal strain to bind corneocytes. Roy et al. (25) identified the A domain as the portion of Aap specifically responsible for adhesion to the host ligand (likely a glycoprotein). Our results corroborated this in the homologous protein SasG, as *S. carnosus* expressing SasG with the A domain mutated did not adhere to corneocytes. As the host ligand is currently unknown, further studies elucidating SasG-mediated binding with the specific SC ligand are warranted upon its discovery. The importance of CWA proteins in adhesion suggests that using inhibitors of these binding interactions could be a promising therapeutic approach to prevent S. aureus skin colonization in individuals at risk for infection.

There are some limitations to this method in terms of quantification and qualitatively in terms of corneocyte staining. In this assay, quantification with Fiji ImageJ acquires the percent area of the entire image, even though corneocytes do not fill 100% of the image. However, there may be other ways in which to utilize ImageJ that may fill this gap. For the trypan blue staining, we were unable to acquire images that accurately represented bacterial adhesion after blocking with protein. It may be that incubation and subsequent washing for protein, bacteria, and staining caused bacteria to wash off after the final phosphate-buffered saline (PBS) wash. This assay can also result in nonnormal distribution, as demonstrated in Fig. 3A. Collecting corneocytes from the skin of different volunteers may contribute to this, as the thickness and distribution of the corneocytes can differ depending on the individual and skin condition. Despite these limitations, this method provides a useful way to study adhesion of staphylococci to corneocytes. This is especially beneficial for comparing trends of adhesion of different species and strains and/or with the use of *S. carnosus* as a model surrogate organism. This method has the potential to be used in studies with disease-state skin corneocytes (such as those from AD skin), nasal corneocytes, and animal corneocytes and with other fluorescently labeled microorganisms.

## MATERIALS AND METHODS

### Corneocyte collection.

Collection of corneocytes was optimized based on the method published by Roy et al. (25), with modifications. Healthy corneocytes were collected from either the lower or upper arm near the elbow from human volunteers. Skin that was not overly moist (had not been recently moisturized) and with little to no hair was chosen so that a more confluent layer of corneocytes could be collected (the condition of the skin may alter with the regional climate). Approximately six or seven discs can be applied to the upper or lower arm near the elbow at a time per volunteer to stay near the elbow. The selected area of skin was cleaned with an alcohol wipe and allowed to air dry. Clear, adhesive tape stripping discs (d-Squame D100; Clinical & Derm) were applied to the cleaned area (a different area of skin for each disc) and pressed firmly onto the skin for 30 s with a pressure device (d-Squame D500; Clinical & Derm) to ensure an even coating of cells on the disc. The discs with collected corneocytes were then carefully removed, taking care not to touch the clear adhesive area of the disc to prevent contamination and abrasion. Collection was considered successful if a thick coating of skin cells was perceptible. A sterile, empty pipette tip box and rack served as a moist chamber with a thin layer of tap water to maintain humidity. The discs with corneocytes were then placed adhesive side up on the empty tip box rack, and the lid was lightly closed to prevent possible settling of contaminants in the air onto the corneocytes. The corneocytes were used within an hour after collection and stored at ambient temperature in the tip box chamber until addition of bacterial/protein solutions.

### Staphylococcal strains and plasmids.

Several species and strains of Staphylococcus were used in this study in order to compare adhesion of different MRSA strains and CoNS. The strains used in this study are listed in Table 1. All strains except the *S. carnosus* strains expressed GFP via maintenance of plasmid pCM29 with constitutively expressed superfolder green fluorescent protein (sGFP) (38). The plasmids were electroporated into recipient strains (39, 40).

To construct GFP-expressing *S. carnosus* strain AH5687 (a derivative of the strain TM300 [41]), homologs of S. aureus ClfB and protein A, which could potentially interfere with the adhesion assays, were mutated. SCA_2283, a homolog of S. aureus ClfB, was replaced with an expression construct of sGFP driven by a *sarA* P1 promoter. Fragments of 1,000 bp upstream and downstream of *SCA_2238* were amplified with primer pairs JK131/JK132 and JK135/JK136 (primer sequences are provided in Table 2). sGFP was amplified from pCM29 (38) with primers JK133/JK134. The resulting fragments were cloned into pIMAY (42) using Gibson Assembly, producing pJK17. pJK17 was electroporated into *S. carnosus* TM300 using Escherichia coli DC10B (42) as an intermediate cloning host. Allelic replacement of *SCA_2238* sGFP resulted in *S. carnosus* TM300 Δ*SCA_2238*::sGFP. Fragments of 1,000 bp upstream and downstream of *SCA_2092*, a gene encoding a homolog of S. aureus protein A, were amplified with primer pairs JK142/JK143 and JK144/JK145 and cloned into pIMAY using Gibson Assembly, producing pJK18. pJK18 was electroporated into *S. carnosus* TM300 Δ*SCA_2238*::sGFP, with E. coli DC10B used as an intermediate cloning host, and the gene was deleted using allelic exchange (42). Presence of desired mutations in both genes were confirmed via PCR, and the resulting strain, *S. carnosus* TM300 Δ*SCA_2238*::sGFP Δ*SCA_2092*, was named *S. carnosus* AH5687.

**TABLE 2:** Oligonucleotides used in this study

Code	Name	Sequence
HC416	MW2 SasG 5′ KpnI	5′-GTTGGTACCCACTGTAAGTAAAGTGGAAAATATGGAA-3′
HC417	MW2 SasG 3′ SacI	5′-GTTGAGCTCTAGGTCTTTCAATCCAACTTTTGG-3′
HC527	MW2 SasG Δ*A domain* b-g-L-II_R	5′-GTCAGATCTAGCTGCTTCTGCCTCTTGTTGAG-3′
HC528	MW2 SasG Δ*A domain* b-g-L-II_F	5′-GTCAGATCTCTAACGCCTAATCTAGTTAATAAAACAGA-3′
JK131	SCA2283_upstream_F	5′-ACTAAAGGGAACAAAAGCTGGGTACGGCTTTTAATAAATCAAGATGC-3′
JK132	SCA2283_upstream_R	5′-CAAAAATATCAGAAAATCCCTCCTAAGATATAAAC-3′
JK133	Psar_sGFP_F	5′-TAGGAGGGATTTTCTGATATTTTTGACTAAACCAAATGC-3′
JK134	Psar_sGFP_R	5′-TATTTAAATGATTTTAGTGGTGGTGGTGGTG-3′
JK135	SCA2283_downstream_F	5′-CACCACCACTAAAATCATTTAAATAGAGGGCTG-3′
JK136	SCA2283_downstream_R	5′-ACTCACTATAGGGCGAATTGGAGCTGGTTTAATTTGCAGATATTCAC-3′
JK142	SCA2092_upstream_F	5′-ACTAAAGGGAACAAAAGCTGGGTACCGATACTATGTATAAAGTAATTGTATTTGATG-3′
JK143	SCA2092_upstream_R	5′-AATAAGTTTGTAGATGTGAAATCCTCCTATTG-3′
JK144	SCA2092_downstream_F	5′-AGGATTTCACATCTACAAACTTATTCTACTCTTACC-3′
JK145	SCA2092_downstream_R	5′-ACTCACTATAGGGCGAATTGGAGCTCACAATTTGGGTTTATGCAATTATTTATTTC-3′
KM22	COL SasG 3′ SacI	5′-GTTGAGCTCGAATGCATCCACCAGCTTTT-3′

SasG-expressing *S. carnosus* strains were generated by electroporation of tetracycline-inducible plasmid pALC2073 encoding SasG driven by the TetR-controlled P*_xyl_*/*_tetO_* promoter into AH5687 (43, 44). An empty vector (EV) strain containing pALC2073 was electroporated into AH5687, resulting in strain AH5907. To construct AH5905, 1,000-bp fragments upstream and downstream of SasG from MRSA MW2 (a USA400 strain) were amplified using primers HC416/HC417 (Table 2), cloned into pALC2073 using restriction cloning, and then electroporated into AH5687. This resulted in plasmid pALC2073-*sasG*_MW2_, which promotes expression of full-length SasG from MRSA MW2 in AH5687. To construct AH5908, 1,000-bp fragments upstream and downstream of SasG from MRSA COL were amplified using primers HC416/KM22, cloned into pALC2073 using restriction cloning, and then electroporated into AH5687. This resulted in plasmid pALC2073-*sasG*_COL_, which promotes expression of full-length SasG from MRSA COL in AH5687. To construct AH6012, the entirety of pALC2073-*sasG*_MW2_ except for the A domain region of SasG was amplified using primers HC527/HC528, each containing a b-g-L-II sequence. The resulting plasmid pHC109 (pALC2073-*sasG*_MW2ΔA_) was electroporated into AH5687 and lacks the portion of the protein considered responsible for adhesion to corneocytes in SasG from MRSA MW2. The plasmids were confirmed via PCR.

### Preparation of bacterial cultures.

All strains except *S. carnosus* were grown in tryptic soy broth (TSB; Research Products International) with 10 μg/mL of chloramphenicol to maintain the plasmid (25). *S. carnosus* was grown in TSB without antibiotic. Five milliliters of bacterial cultures was grown in Pyrex Vista rimless 15-mL round-bottom reusable glass culture tubes (Fisher Scientific) at 37°C overnight with shaking aeration at 220 rpm. Overnight cultures were then subcultured 1:50 in 3 mL of TSB with antibiotic (if applicable) and grown to an optical density at 600 nm (OD_600_) of approximately 0.75 at 37°C with shaking aeration. OD was measured using the Genesys 30 VIS spectrophotometer (Thermo Scientific). Cultures were spun down at room temperature for 3 min at 8,000 × *g* using an accuSpin Micro 17 tabletop centrifuge (Fisher Scientific). Cultures were then resuspended in an equal volume of sterile PBS and diluted in PBS to an OD_600_ of 0.15 (~10^7^ CFU/mL) (25). Using this OD results in adhesion of a sufficient number of cells to allow easy enumeration without causing unwanted bacterial clumping (25).

### Preparation of protein solution for adhesion inhibition.

To observe inhibition of adhesion with protein in solution, a BSA control and full-length S. aureus surface protein G (SasG) protein solutions were prepared. BSA was suspended in PBS at a final concentration of 100 μg/mL and filtered using a 0.22-μm syringe filter. Previously characterized, full-length recombinant SasG (purified from MRSA MW2; 250 kDa) was added to PBS at a final concentration of 100 μg/mL (33). The concentration of 100 μg/mL was chosen because it causes significant reduction in adhesion in a dose-response assay (data not shown).

### Incubation (bacteria only).

After collection of corneocytes, 300 μL (~3.3 × 10^6^ CFU total) of prepared staphylococcal suspensions in PBS were individually pipetted slowly as a droplet onto the center of one corresponding disc. The lid was then lightly closed to prevent settling of contaminants in the air onto the discs. The box with the corneocytes was then placed into a humidified 37°C incubator with 5% CO_2_, taking care not to jostle the droplets. The suspension droplets were incubated on the corneocytes for 45 min (25) and prepared for imaging as described in “Corneocyte staining for autofluorescence” and “Imaging.”

### Incubation (inhibition of adhesion with protein followed by bacteria).

Three hundred microliters of prepared BSA or full-length SasG were individually added onto the corneocytes and incubated for 45 min. Corneocytes were then washed and allowed to air dry as described in “Imaging.” Prepared bacterial cultures were then added to the dried corneocytes on the same spot that the protein solution was added to, incubated for 45 min as described in “Incubation (bacteria only),” and then prepared for imaging as described in “Imaging.”

### Corneocyte staining for autofluorescence.

In order to visualize corneocytes fluorescently, trypan blue solution (0.4%; Gibco) was added to corneocytes after addition of bacteria to allow red autofluorescence of the corneocytes. After incubation and washing off the bacteria, corneocytes were allowed to air dry. Three hundred microliters of trypan blue was then added to the dried corneocytes on the area where the bacteria were added and incubated at room temperature for 15 min. The corneocytes were then gently washed twice with 1 mL PBS as described in “Imaging.” The backs of the discs were washed once with 1 mL PBS. Trypan blue was not added to corneocytes if protein was added.

### Imaging.

After incubation and staining, microscope slides (plain glass; 1-mm thickness, 3 by 1 in.; Fisher Scientific) were cleaned with 70% ethanol sprayed on a Kimwipe and allowed to air dry. The corneocytes were washed 10 times with 1 mL PBS. The disc was turned over and washed on the nonadhesive side twice with 1 mL PBS to remove any possible backsplash on the nonadhesive side of the disc. The backs of the discs were blotted gently with a Kimwipe to remove moisture and then placed on one cleaned slide for each disc. The corneocytes were left to air dry on the slides. Once completely dry, a drop of aqueous mounting medium (Glycergel; Agilent Technologies) was placed in the center of each disc. A rectangular coverslip (thickness, 0.14 mm; 22 by 50 mm; Globe Scientific) was then immediately placed onto the disc. The coverslip was secured onto the slide with clear scotch tape. The slides were then stored at 4°C overnight in order for the mounting medium to have adequate time to dry.

The corneocytes were imaged using a Keyence BZ-X 710 fluorescence microscope at ×20 magnification using the bright-field, red, and green channels. Ten images were taken of each disc; each area was marked in the imaging software so as to avoid duplicate images. Overlay images of bright-field and green channels and green and red channels were acquired. The bright-field channel imaged corneocytes only without fluorescence, the green channel imaged green-fluorescent bacteria, the red channel imaged corneocytes with red autofluorescence. Images were not acquired in any areas where there may have been bacterial clumping or disproportionate binding to areas between corneocytes.

### Data and statistical analysis.

Images captured with the green channel were quantified for percent area of adhesion using Fiji ImageJ (45). Images were converted to 8-bit images and adjusted to the Otsu threshold. Once the images were adjusted, the “analyze>measure” prompt in ImageJ was selected to acquire “percent area.” Ten images for each disc/experimental group were statistically analyzed with either one-way analysis of variance (ANOVA) or the nonparametric Kruskal-Wallis equivalent in GraphPad Prism. Ten data points from three independent experiments (*n* = 30) were analyzed for final results in GraphPad Prism. A schematic of the corneocyte adhesion method is shown in Fig. 1.
