# Supplementary material for: Staphylococcal Corneocyte Adhesion: Assay Optimization and Roles of Aap and SasG Adhesins in the Establishment of Healthy Skin Colonization
Source: Microbiol Spectr. 2022 Oct 11;10(6):e02469-22. doi: 10.1128/spectrum.02469-22 (PMC9769725; doi:10.1128/spectrum.02469-22)
Supplement: Supplemental file 1 — Fig. S1. Download spectrum.02469-22-s0001.pdf, PDF file, 0.4 MB [file spectrum.02469-22-s0001.pdf]

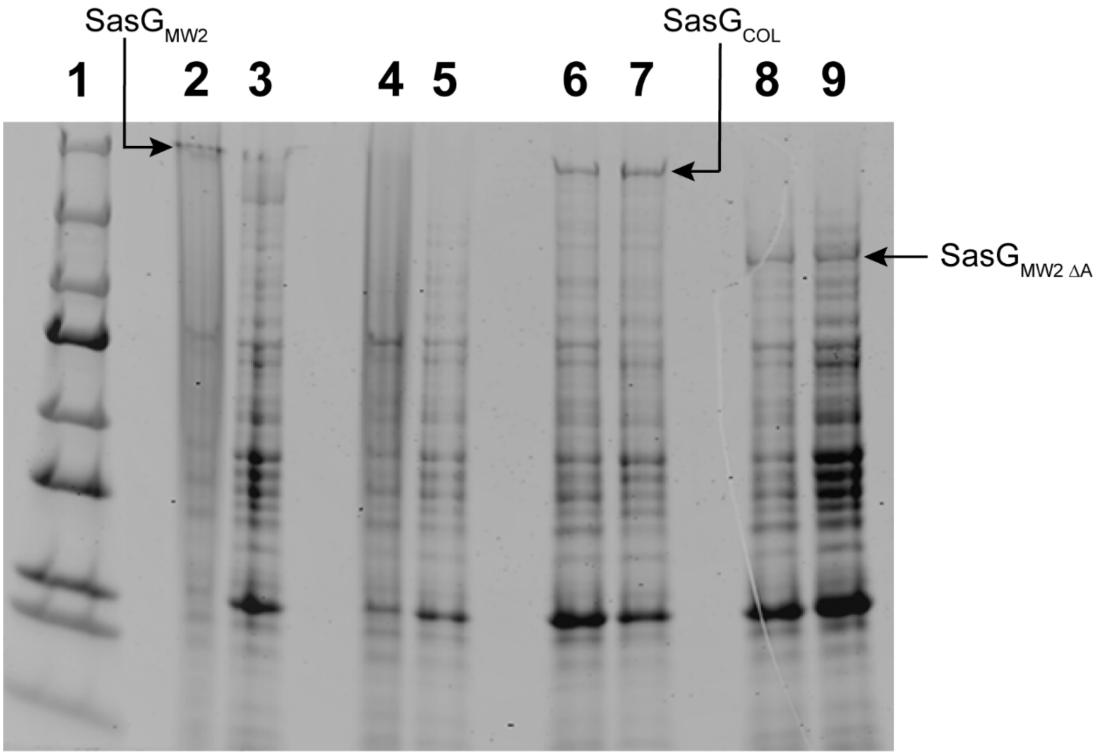

Figure S1. Coomassie stain of *S. carnosus* strains revealing SasG localization. SDS-PAGE electrophoresis followed by Coomassie staining was performed on solubilized proteins from cell wall extracts of *S. carnosus* strains AH5905 (pALC2073-*sasG*$_{MW2}$), AH5907 (pAL2073 EV), AH5908 (pALC2073-*sasG*$_{COL}$), and AH6012 (pALC2073-*sasG*$_{MW2\Delta A}$). Strains were either uninduced or induced with 100 ng/mL anhydrotetracycline. (1) Precision Plus Protein Kaleidoscope 10-250 kDa ladder. (2) AH5905 uninduced. (3) AH5905 induced. (4) AH5907 uninduced. (5) AH5907 induced. (6) AH5908 uninduced. (7) AH5908 induced. (8) AH6012 uninduced. (9) AH6012 induced.
